# Soluble sugar and organic acid composition and flavor evaluation of Chinese cherry fruits

**DOI:** 10.1016/j.fochx.2023.100953

**Published:** 2023-10-21

**Authors:** Jingting Zhou, Shuaiwei Yang, Yan Ma, Zhenshan Liu, Hongxia Tu, Hao Wang, Jing Zhang, Qing Chen, Wen He, Mengyao Li, Yuanxiu Lin, Yunting Zhang, Zhiwei Wu, Yong Zhang, Ya Luo, Haoru Tang, Yan Wang, Xiaorong Wang

**Affiliations:** a College of Horticulture, Sichuan Agricultural University, Chengdu 611130, China; b Key Laboratory of Agricultural Bioinformatics, Ministry of Education, Chengdu 611130, China

**Keywords:** Chinese cherry [*Cerasus pseudocerasus* (Lindl.) G.Don], Soluble sugars, Organic acids, HPLC, Fruit favor, Rating criteria, Glucose (PubChem CID: 5793), Fructose (PubChem CID: 5984), Sorbitol (PubChem CID: 5780), Sucrose (PubChem CID: 5988), Malic acid (PubChem CID: 525), Citric acid (PubChem CID: 311), Quinic acid (PubChem CID: 6508), Succinic acid (PubChem CID: 1110), Lactic acid (PubChem CID: 612), Shikimic acid (PubChem CID: 8742)

## Abstract

Chinese cherry is an economically important fruit crop native to China. Flavor quality is greatly influenced by compositions of soluble sugars and organic acids. To better understand the flavor quality of Chinese cherry, we determined sugar and acid components in thirty-eight landrace and cultivar collections, and two wild resources using the HPLC method. Glucose and fructose were the main components, accounting for 85.91% of soluble sugars. Malic acid was the predominant organic acid, with an average proportion of 65.73% of total acids. Correlation and PCA analysis revealed seven key indicators for evaluating fruit flavor. Compared with wild Chinese cherry, the cultivated collections exhibited higher levels of soluble sugars, especially fructose, and lower levels of organic acid, particularly malic acid in fruits. Finally, we have established grading criteria for seven flavor indicators in Chinese cherry. Our study provides valuable references for identifying flavor compounds and improving flavor quality of Chinese cherry.

## Highlights

• Fructose and glucose were the predominant sugars in Chinese cherry fruits.
• Malic acid was the most abundant organic acid in Chinese cherry fruits.
• Increase in fructose and reduction in malic acid during domestication.
• Rating criteria of flavor evaluation indicators was established.

## 1 Introduction

Chinese cherry [*Cerasus pseudocerasus* (Lindl.) G.Don], native to China, belongs to the genus *Cerasus* in the family Rosaceae and has a cultivation history of 3,000 years (Yü, Lu, Ku, Li & Chen, 1986). It is one of the four major fruiting cherry species in the world, along with sweet cherry (*C. avium*), sour cherry (*C. vulgaris*), and nanking cherry (*C. tomentosa*) (Huang, Wang, Chen, Chen & Tang, 2013), and has gained increasing significance in China’s cherry industry. During the long period of domestication and selection, Chinese cherry landraces adapted to diverse local ecological and climatic regions have been widely planted across China, and the soluble sugars and organic acids contents and fruit flavor have remarkably varied (Chen et al., 2016, Huang et al., 2013). In addition to fruit color, shape and size, the landraces and cultivars demonstrate great variation and rich diversity in physiological characteristics that determine key fruit quality attributes, especially flavor, including sugar and acid compositions.

Flavor is a crucial element determining food qualities, and it generally affects consumers’ perception and preference (Shu, Chen, Sun, Cao, Liu & Xu, 2022). Food flavor is a very complex property, involving sugars, organic acids, amino acids and many other secondary metabolites (Li et al., 2023). In terms of fruit foods, fruit flavor is greatly influenced by the composition of soluble sugars and organic acids (Gonçalves, Campos, Alves, Garcia-Viguera, Moreno & Silva, 2021). Soluble sugars and organic acids are not only nutrients but also critical indicators of the quality and taste of fruit crops (Blando & Oomah, 2019). With an improvement in people’s living standards, fruit breeders, marketers, consumers, and processors are increasingly focused on the fruit falvor, especially the sweet and sour taste of fruits, as well as the improvement of fruit flavor (Cao, Jiang, Lin, Li, Sun & Chen, 2015; Ma et al., 2015). The composition of soluble sugars and organic acids exhibits great differences among fruit crops. Fruits can be roughly divided into three types according to the predominant soluble sugar at fruit ripening stages, including fructose accumulation type (i.e., apple, strawberry) (Li et al., 2021, Akhatou and Fernández, 2014), sucrose accumulation type (i.e., plum, apricot) *(*Bae, Yun, Yoon, Nam, Kwon & Jun 2014) and glucose accumulation type (sweet cherry) (Gonçalves, Campos, Alves, Garcia-Viguera, Moreno & Silva, 2021). Based on the type of the most abundant organic acid, fruits can be divided into two main types: malic acid dominant type (Li et al., 2021, apple; Usenik, Fabčič & Štampar, 2008, sweet cherry) and citric acid dominant type (Wang et al., 2021, citrus). So far, there have been few studies on the soluble sugar and organic acid composition of Chinese cherry fruits. The patterns of soluble sugar and organic acid accumulation in Chinese cherry fruits still remain unclear.

Soluble sugars and organic acids are among the phenotypes selected by humans during fruit domestication (Cornille, Giraud, Smulders, Roldán-Ruiz & Gladieux, 2014). Recent studies have found that the acidity of the fruit rather than the sweetness may have been selected during the domestication of citrus (Wang et al., 2021) and apple (Ma et al., 2015), while both the fruit’s acidity and sweetness have undergone significant selection in cultivated peach (Li et al., 2019). Therefore, a comprenhensive understanding of the characteristics of fruit sugar and acid is essential for better utilization of these natural resources in breeding to improve fruit quality. Cultivated Chinese cherry is characterized by large, red-colored fruits with a rich flavor, while wild cherry exhibits small, yellowish fruits with a sour flavor (Table S1, Fig. S1). However, the characteristics of fruit sugars and acids during domestication histrory are unknown.

Currently, HPLC, GC–MS and LC-MS have been successfully and widely used to determine food flavor chemicals, which have provided valuable information on the characteristic metabolites for variety identification and the improvement of food quality (Zhang et al., 2020, Lin et al., 2023, Li et al., 2023). Especially, metabolomics analysis technology dedicated to more fine metabolites (Fraga-Corral, Carpena, Garcia-Oliveira, Pereira, Prieto & Simal-Gandara, 2022; Pinu, 2015). Nevertheless, HPLC still exhibits the high efficency and convenience in determining the food flavor, particularly the fruits flavor, characterized by soluble sugar and organic acid compounds (Shu et al., 2022). In the present study, based on the field invesigation and collections (Chen et al., 2016), we selected thirty-eight Chinese cherry landrace and cultivar collections and two wild accessions as representative materials to determine the sugar and acid components using the HPLC method. Our objectives were (i) to characterize the sugar and acid components and contents of different Chinese cherry fruits; (ii) to evaluate the effect of sugar and acid composition on fruit flavor; (iii) to explore the changes in sugar and acid during the domestication process; and (iv) to establish rating criteria for fruit flavor evaluation indicators of Chinese cherry. The results can provide a basis for the evaluation of fruit flavor and genetic improvement of fruit quality in Chinese cherry.

## 2 Materials and methods

### 2.1 Plant materials

In this study, a total of forty Chinese cherry accessions were analyzed. Among them, thirty-eight were landraces and cultivars, respresenting the distribution and diversity of cultivated Chinese cherry in China. Two wild resourcees from the original regions of cultivated Chinese cherry were also included (Chen et al., 2016, Huang et al., 2013, Zhang et al., 2021). The detailed information about these accessions is provided in Table S1 and Fig. S1. All these accessions were preserved in the Cherry Germplasms Repository of Sichuan Province at the modern agricultural research and development base in Sichuan Agricultural University. They were grafted onto wild Chinese cherry rootstock and cultivated under a rain shelter with normal field conditions, including irrigation, fertilization, and disease and pest control. In 2022, approximately 100 fruits with similar color and uniform size were sampled for each accession, immediately frozen in the liquid, and store at − 80 °C for subsequent analysis.

### 2.2 Sensory evaluation

Two kilograms of fruit were supplied per accession for sensory analysis. A ten-trained panel evaluated the taste of the studied Chinese cherry accessions in the laboratory according to the method of Karagiannis with modifications (Karagiannis et al., 2021). Based on subjective feelings, the tasters first determined the flavor rating (sour, sour–sweet, sweet–sour, sweet) and then scored according to the score range of the rating (sour, 1.0 ∼ 2.5; sour–sweet, 2.6 ∼ 5.0; sweet–sour, 5.1 ∼ 7.5; sweet, 7.6 ∼ 10.0). The final score was calculated with the formula, the final score* = (total score - the highest score - the lowest score)/ 8. The final scores were shown in Table S1.

### 2.3 Determination of soluble solid, soluble sugar and titratable acid contents

Total soluble solid and titratable acid contents were measured using a refractometer (Pocket Brix-Acidity Meter Master Kit, ATAGO, Tokyo, Japan) through the refractometer method and electroconductivity method, respectively. Fresh fruits were used for the determination of soluble solids and titratable acids. We used the undiluted juice to measure soluble solids. To measure acid, 0.50 g of fruit juice was collected and diluted to 25.00 g using purified water. Three independent biological replicates were used for each accession.

The soluble sugar content was detected by the anthrone colorimetric method (Hou, 2015). Firstly, 0.2 g fresh fruit was ground to powder in liquid nitrogen. Four mL of distilled water was added to the powder and the extract was boiled in boiling water for 30 min. Then, the extract was centrifuged at 12,000 g and 4 °C for 20 min, and the supernatant was extracted. Another round of supernatant extraction was conducted using the precipitation according to the aforementioned process. The supernatant from the two rounds of extraction was combined and fixed to 8 mL with distilled water. Finally, 1 mL extracting solution was mixed with 1 mL distilled water, 0.5 mL anthrone ethyl acetate and 5 mL concentrated sulphuric acid, and the soluble soluble sugar content was calculated by absorbance at 630 nm using the Thermo Scientific Microplate Reader (Multiskan GO). Three independent biological replicates were used for each accession.

### 2.4 Extraction and determination of sugar and acid contents

The extraction, detection, and quantification of sugars and organic acids were performed according to Ma’s method (Ma et al., 2015) with slight modifications. Fresh samples were completely freeze-dried in a vacuum environment of −80 °C using a freeze dryer (EYELA FDU-2110, Japan). The dry sample was ground into powder in liquid nitrogen. Approximately 0.5 g of powder were extracted for 30 min in ultrasound with 10 mL of ultrapure water. The suspension was centrifuged at 10,000 g for 10 min at 4℃. Finally, the supernatant from each sample was brought to 10 mL, and 1 mL was taken, then filtered with a 22-μm microporous membrane for HPLC (high-performance liquid chromatography) analysis.

The filtered supernatants were used to measure sugar and organic acids using an Agilent 1260 Infinity II. Sugars were detected by an Agilent G1362A refractive index detector (RID) with a reference cell maintained at 40 °C. A YMC-Pack Polyamine II column (4.6 mm × 250 mm, 5 μm) was used, with the column maintained at 30 °C. The mobile phase consisted of acetonitrile: ultrapure water = 80:20 (v/v), and the flow rate was set at 0.6 mL·min^−1^. The injection volume was 10 μL. Organic acids were detected using an Agilent G1314F Variable Wavelength detector (VWD). A Restek Allure Organic Acid column (4.6 mm × 350 mm, 5 μm) was used, with the column maintained at 20 °C. The mobile phase consisted of 5 % methanol and 95 % phosphoric acid solution. The flow rate was set at 0.6 mL·min^−1^. UV absorbance was detected at 210 nm. Sugar and organic acid contents were expressed as g·kg^−1^ dry weight (DW).

### 2.5 Data analysis

Box-plots and pie charts of sugar and organic acid composition were generated using Origin 2022 (OriginLab, USA). The results of correlation and principal component analysis were conduted and visualized using the Chiplot online website (https://www.chiplot.online). All statistical analyses were performed using SPSS27.0 (IBM, Armonk, NY, USA) software. Significant differences were estimated using two-way ANOVA with Duncan’s multiple range tests (*p* < 0.05). The Shapiro-Wilk test was used to test the normality of the indices. The 10th, 30th, 70th, and 90th percentiles of the normal distribution curve were used as the graded nodal values of the index (Zhang et al., 2020).

## 3 Results and discussion

### 3.1 Composition of soluble sugars in cultivated Chinese cherry fruits

Soluble solid content (SSC) is one of the most important factors in determining the intrinsic quality of fresh fruit. SSC is closely related to sugar and directly impacts consumer acceptability (Serradilla, Hernández, López-Corrales, Ruiz-Moyano, de Guía Córdoba & Martín, 2016). The SSC varied considerably among 38 cultivated Chinese cherry landraces (Table S1), ranging from 13.20 % (YJ) to 27.53 % (ZaZ4). The mean SSC was 16.37 %, which is generally consistent with our previous report (Wang et al., 2022). The total soluble sugar content ranged from 381.33 to 698.62 g·kg^−1^ DW with an average value of 563.52 g·kg^−1^ DW (Table 1).

**Table 1:** Components of soluble sugars in cultivated Chinese cherry fruits.

Accession	Soluble sugar content	Glucose		Fructose		Sorbitol		Sucrose	
		Content	Proportion	Content	Proportion	Content	Proportion	Content	Proportion
ZaZ8	477.49 ± 52.36	176.72 ± 9.14	37.01	198.84 ± 5.64	41.64	62.39 ± 5.87	13.07	ND	–
BJ2	480.70 ± 59.32	173.50 ± 20.75	36.09	201.07 ± 10.82	41.83	36.54 ± 7.00	7.60	5.00 ± 0.46	1.04
BJ4	619.23 ± 110.54	249.08 ± 16.62	40.22	271.66 ± 24.22	43.87	32.34 ± 5.59	5.22	7.94 ± 2.15	1.28
BJ6	625.01 ± 79.86	250.43 ± 40.71	40.07	260.52 ± 39.71	41.68	56.30 ± 9.69	9.01	5.18 ± 0.73	0.83
BJ7	553.52 ± 65.20	208.03 ± 10.87	37.58	219.54 ± 30.73	39.66	33.52 ± 4.74	6.06	8.05 ± 0.45	1.45
FM2	668.21 ± 122.15	344.79 ± 4.77	51.60	275.55 ± 7.09	41.24	31.23 ± 5.68	4.67	6.66 ± 0.38	1.00
GY1	596.71 ± 90.87	268.73 ± 17.68	45.03	272.37 ± 35.50	45.65	33.21 ± 5.78	5.57	11.52 ± 1.61	1.93
GY2	555.84 ± 70.45	264.42 ± 24.77	47.57	222.19 ± 17.09	39.97	52.31 ± 2.92	9.41	5.27 ± 1.55	0.95
HC	381.33 ± 36.98	187.76 ± 10.98	49.24	124.10 ± 12.56	32.54	53.12 ± 0.47	13.93	6.80 ± 1.18	1.78
HeF	541.56 ± 48.66	219.97 ± 35.92	40.62	209.08 ± 33.65	38.61	25.55 ± 5.77	4.72	5.99 ± 0.44	1.11
HZ1	512.21 ± 70.15	194.05 ± 34.46	37.88	213.99 ± 12.93	41.78	34.58 ± 7.68	6.75	13.52 ± 1.22	2.64
HZZ	498.71 ± 59.66	184.83 ± 34.91	37.06	198.72 ± 19.56	39.85	24.23 ± 2.62	4.86	8.21 ± 0.59	1.65
JY4	619.86 ± 89.65	288.26 ± 24.32	46.50	276.79 ± 16.49	44.65	40.79 ± 5.22	6.58	5.83 ± 1.01	0.94
LQ	583.31 ± 67.45	242.92 ± 22.52	41.65	261.40 ± 23.20	44.81	58.55 ± 9.83	10.04	9.55 ± 2.09	1.64
LYg	563.39 ± 81.05	259.95 ± 40.23	46.14	262.37 ± 28.71	46.57	23.21 ± 2.39	4.12	4.19 ± 0.12	0.74
LYi5	504.77 ± 67.48	224.43 ± 18.21	44.46	253.36 ± 15.16	50.19	14.52 ± 4.92	2.88	7.18 ± 1.14	1.42
MY3	665.33 ± 107.58	366.54 ± 36.41	55.09	214.94 ± 25.44	32.31	54.34 ± 5.68	8.17	7.31 ± 1.27	1.10
MY5	600.51 ± 98.35	283.74 ± 39.75	47.25	248.48 ± 32.32	41.38	33.39 ± 6.01	5.56	8.24 ± 0.12	1.37
MZ3	514.85 ± 82.59	189.61 ± 3.22	36.83	211.49 ± 38.66	41.08	40.14 ± 8.10	7.80	6.17 ± 1.04	1.20
NZH	490.64 ± 64.29	204.48 ± 34.83	41.68	209.14 ± 36.94	42.63	34.90 ± 17.50	7.11	7.97 ± 0.17	1.62
PD3	596.69 ± 87.45	280.58 ± 32.26	47.02	255.84 ± 18.60	42.88	42.49 ± 7.09	7.12	9.94 ± 0.36	1.67
PJHH	698.62 ± 108.96	343.73 ± 13.40	49.20	302.17 ± 32.60	43.25	36.66 ± 3.08	5.25	4.99 ± 0.25	0.71
SP4	580.61 ± 84.32	262.31 ± 24.27	45.18	258.23 ± 16.53	44.48	44.94 ± 8.62	7.74	7.05 ± 0.72	1.21
TH2	589.07 ± 89.20	261.86 ± 13.21	44.45	286.44 ± 28.26	48.63	28.55 ± 4.04	4.85	6.99 ± 1.02	1.19
WN1	536.69 ± 49.54	225.52 ± 38.89	42.02	224.99 ± 14.72	41.92	45.46 ± 5.10	8.47	8.47 ± 0.35	1.58
XC1	638.92 ± 64.89	284.16 ± 7.07	44.47	268.49 ± 36.45	42.02	45.54 ± 0.12	7.13	6.26 ± 0.82	0.98
XC2	582.76 ± 75.69	301.68 ± 21.87	51.77	239.00 ± 4.45	41.01	40.97 ± 7.20	7.03	ND	–
YJ	489.01 ± 61.21	219.28 ± 39.25	44.84	197.49 ± 30.56	40.39	46.17 ± 10.76	9.44	6.34 ± 0.88	1.30
YL2	607.82 ± 101.54	261.51 ± 35.75	43.02	270.18 ± 31.27	44.45	39.98 ± 8.79	6.58	5.96 ± 0.89	0.98
YX6	592.77 ± 78.69	295.54 ± 45.02	49.86	245.48 ± 27.84	41.41	17.16 ± 2.75	2.89	ND	–
ZT1	517.54 ± 75.21	226.21 ± 34.79	43.71	234.30 ± 43.34	45.27	22.14 ± 4.40	4.28	4.93 ± 0.39	0.95
ZY3	524.46 ± 68.23	192.32 ± 23.16	36.67	206.65 ± 32.82	39.40	28.85 ± 3.12	5.50	18.09 ± 1.72	3.45
ZaZ4	697.57 ± 136.32	294.16 ± 31.17	42.17	294.81 ± 36.96	42.26	37.07 ± 1.40	5.31	8.04 ± 1.30	1.15
ZeZ6	428.44 ± 45.69	214.28 ± 10.53	50.01	142.97 ± 24.05	33.37	28.57 ± 2.16	6.67	4.87 ± 0.16	1.14
ZaZ6	473.56 ± 50.23	214.90 ± 28.63	45.38	189.58 ± 5.10	40.03	56.54 ± 2.76	11.94	ND	–
ZeZ9	589.15 ± 78.95	265.12 ± 32.47	45.00	212.54 ± 37.00	36.08	40.41 ± 1.86	6.86	5.09 ± 0.85	0.86
BJ7-2	672.96 ± 115.23	311.50 ± 11.90	46.29	291.24 ± 22.81	43.28	42.59 ± 10.55	6.33	21.18 ± 4.09	3.15
HF	543.87 ± 66.89	261.03 ± 43.89	47.99	237.50 ± 32.74	43.67	24.29 ± 2.27	4.47	6.31 ± 0.82	1.16
Mean	563.52	249.95	44.18	235.88	41.73	37.99	6.87	7.80	1.39
SD	72.92	48.62	4.76	39.81	3.77	11.76	2.50	3.61	0.62
CV(%)	13.44	19.45	10.78	16.88	9.04	30.95	36.45	46.32	44.84

Note: ND, not detected. Content (g·kg^−1^ DW), Proportion (%).

**Fig. 1:**
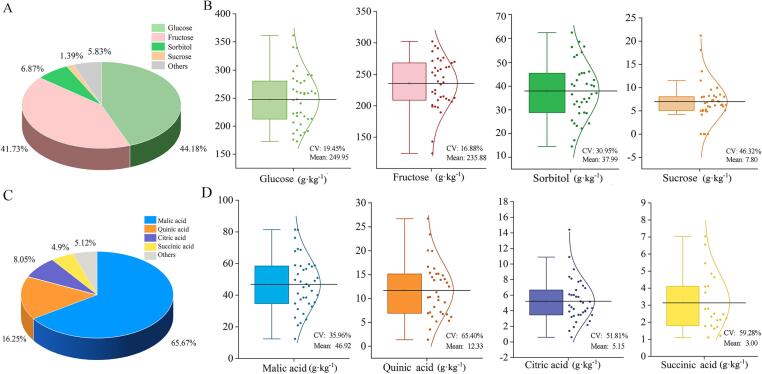
Proportion (A, C) and distribution (B, D) of sugar and acid components in 38 cultivated Chinese cherry accessions. Others in A indicate the sugar components except for glucose, fructose, sorbitol and sucrose within soluble sugar. Others in C indicate tartaric, lactic, oxalic, ascorbic, shikimic, maleic, and fumaric acid. The horizontal lines in the interior of the box are mean values. Each scatter indicates the content of each material. The line on the right shows the fitted distribution curve. CV, coefficient of variation.

In Chinese cherry fruits, we identified four soluble sugars (glucose, fructose, sorbitan, and sucrose), accounting for 94.17 % of the soluble soluble sugars (Fig. 1A). Among the four soluble sugars, glucose and fructose are two main components, accounting for 44.18 % and 41.73 % of the soluble sugars, respectively (Fig. 1A). Glucose content ranged from 154.83 to 463.73 g·kg^−1^ DW, and fructose content was between 142.97 and 338.17 g·kg^−1^ DW. By contrast, glucose occupied a relatively high proportion (51 % − 60 %) of soluble sugar contents and fructose only account for a low proportion (30 % − 40 %) in other fruiting cherries, such as sweet cherry (*Cerasus avium*) and sour cherry (*Cerasus vulgaris*) (De Leo et al., 2021). The remaining sugars (others) only account for 5.83 % of soluble sugar contents (Fig. 1A).

It is well known that sorbitol is greatly benificial for human health, including diuresis promotion and blood pressure reduction (Lee, 2015). In this study, sorbitol was detected in all examined Chinese cherry accessions, with a content of 14.52 to 62.59 g·kg − 1 DW and an average proportion of 6.87 %. Meantime, sucrose content ranged from 4.19 to 23.18 g·kg − 1 DW in Chinese cherry fruits. These results indicate the high health care and nutritional value of Chinese cherry fruits.

Among the 38 examined accessions, the coefficient of variation (CV) of glucose and fructose was 19.45 % and 16.88 %, respetively. The data showed a low coefficient of variation, indicating the stability of the main sugar components in Chinese cherry fruits. Even the fructose, the major contributor to the sweetness of the fruit flavor, was relatively stable.

### 3.2 Composition of organic acids in cultivated Chinese cherry fruits

Organic acids play a crucial role in the development of fruit flavor, directly influencing the overall flavor quality (Blando & Oomah, 2019). In Chinese cherry fruit, eleven organic acid components were identified (Table 2, Fig. 1C, D). The most abundant component was malic acid, accounting for an average proportion of 65.73 % and up to 91.42 % of the total acid content. The malic acid was overwhelmingly dominant in Chinese cherry fruits, which was consistent with the reports of other fruiting cherries (Blando & Oomah, 2019; Chockchaisawasdee, Golding, Vuong, Papoutsis & Stathopoulos, 2016).

**Table 2:** Components of organic acids in cultivated Chinese cherry fruits.

Accession	Malic acid		Quinic acid		Citric acid		Succinic acid		Others (^1^tartaric, ^2^lactic, ^3^oxalic, ^4^ascorbic, ^5^shikimic, ^6^maleic and ^7^fumaric acid)	
	Content	Pro	Content	Pro	Content	Pro	Content	Pro	Content	Pro
ZaZ8	49.66 ± 2.22	63.18	20.05 ± 1.57	25.51	4.69 ± 0.40	5.97	2.77 ± 0.14	3.52	^1^0.03 ± 0.02, ^2^1.31 ± 0.09, ^3^0.09 ± 0.02	^1^0.04, ^2^1.67, ^3^0.11
BJ2	41.04 ± 1.87	71.26	6.91 ± 0.13	12.00	6.10 ± 0.09	10.59	1.77 ± 0.04	3.07	^2^1.67 ± 0.15, ^3^0.10 ± 0.01	^2^2.90, ^3^0.17
BJ4	34.71 ± 7.59	62.09	16.62 ± 0.24	29.73	3.90 ± 0.88	6.98	ND	–	^2^0.62 ± 0.02, ^3^0.05 ± 0.01	^2^1.11, ^3^0.09
BJ6	25.80 ± 5.82	63.16	7.08 ± 1.49	17.33	3.20 ± 0.98	7.83	4.09 ± 0.79	10.01	^2^0.68 ± 0.19	^2^1.66
BJ7	20.90 ± 0.78	62.30	6.22 ± 0.20	18.54	4.45 ± 0.19	13.26	1.11 ± 0.09	3.31	^2^0.81 ± 0.02, ^3^0.06 ± 0.01	^2^2.41, ^3^0.18
FM2	58.86 ± 4.33	75.93	10.22 ± 0.60	13.18	3.34 ± 0.34	4.31	3.74 ± 0.31	4.82	^2^1.36 ± 0.11	^2^1.75
GY1	81.39 ± 11.33	89.30	ND	–	2.54 ± 0.56	2.79	6.56 ± 0.15	7.20	^3^0.58 ± 0.04, ^4^0.07 ± 0.01	^3^0.64, ^4^0.08
GY2	12.39 ± 2.58	60.85	ND	–	4.23 ± 0.91	20.78	2.79 ± 0.11	13.70	^2^0.95 ± 0.17	^2^4.67
HC	67.78 ± 6.35	76.84	10.30 ± 0.50	11.68	6.00 ± 1.59	6.80	ND	–	^1^0.88 ± 0.02, ^2^2.93 ± 0.05, ^3^0.29 ± 0.08, ^7^0.03 ± 0.01	^1^1.00, ^2^3.32, ^3^0.33, ^7^0.03
HeF	48.59 ± 0.81	68.71	14.45 ± 0.13	20.43	6.03 ± 0.66	8.53	ND	–	^2^1.03 ± 0.07, ^3^0.53 ± 0.02, ^6^0.09 ± 0.01	^2^1.46, ^3^0.75, ^6^0.13
HZ1	45.06 ± 5.98	83.69	ND	–	5.81 ± 0.84	10.79	ND	–	^1^1.54 ± 0.17, ^2^0.94 ± 0.15, ^3^0.35 ± 0.05, ^5^0.14 ± 0.03	^1^2.86, ^2^1.75, ^3^0.65, ^5^0.26
HZZ	31.18 ± 1.64	55.07	13.84 ± 0.38	24.44	8.37 ± 0.42	14.78	2.33 ± 0.08	4.12	^2^0.90 ± 0.04	^2^1.59
JY4	22.35 ± 1.18	60.60	8.21 ± 0.60	22.26	1.42 ± 0.06	3.85	4.11 ± 0.19	11.14	^1^0.11 ± 0.01, ^3^0.23 ± 0.01, ^4^0.45 ± 0.01	^1^0.30, ^3^0.62, ^4^1.22
LQ	28.15 ± 2.87	64.61	5.18 ± 0.16	11.89	6.66 ± 0.99	15.29	ND	–	^2^6.58 ± 0.47	^2^8.22
LYg	53.27 ± 7.05	65.32	11.30 ± 1.62	13.86	14.4 ± 0.34	17.66	2.50 ± 0.05	3.07	^3^0.08 ± 0.01	^3^0.10
LYi5	37.28 ± 4.86	65.51	9.66 ± 0.94	16.97	8.13 ± 1.04	14.29	1.80 ± 0.10	3.16	^7^0.04 ± 0.01	^7^0.07
MY3	58.41 ± 10.92	80.82	10.35 ± 1.39	14.32	3.47 ± 1.55	4.80	ND	–	^4^0.04 ± 0.02	^4^0.06
MY5	55.94 ± 3.61	71.99	15.39 ± 0.86	19.80	5.77 ± 0.32	7.43	ND	–	^2^0.44 ± 0.07, ^3^0.17 ± 0.01	^2^0.57, ^3^0.22
MZ3	69.22 ± 4.20	77.75	13.89 ± 0.64	15.60	3.48 ± 0.15	3.91	2.11 ± 0.07	2.37	^4^0.33 ± 0.03	^4^0.37
NZH	38.44 ± 1.50	57.11	14.82 ± 2.24	22.02	10.90 ± 0.11	16.19	1.63 ± 0.02	2.42	^1^0.78 ± 0.01, ^2^0.30 ± 0.01, ^3^0.36 ± 0.03, ^4^0.08 ± 0.01	^1^1.16, ^2^0.45, ^3^0.53, ^4^0.12
PD3	81.27 ± 6.51	84.33	ND	–	5.21 ± 0.30	5.41	2.15 ± 0.17	2.23	^1^4.82 ± 0.42, ^2^2.31 ± 0.29, ^3^0.39 ± 0.03, ^4^0.14 ± 0.02, ^7^0.08 ± 0.01	^1^5.00, ^2^2.40, ^3^0.40, ^4^0.15, ^7^0.08
PJHH	24.68 ± 2.45	68.88	7.16 ± 1.11	19.98	3.79 ± 0.76	10.58	ND	–	^3^0.20 ± 0.01	^3^0.56
SP4	56.60 ± 2.52	68.25	14.05 ± 1.14	16.94	9.32 ± 0.51	11.24	2.46 ± 0.12	2.97	^3^0.37 ± 0.01, ^5^0.13 ± 0.01	^3^0.45, ^5^0.16
TH2	38.46 ± 1.81	71.37	6.75 ± 0.11	12.53	7.72 ± 0.09	14.33	ND	–	^2^0.94 ± 0.06, ^7^0.02 ± 0.00	^2^1.74, ^7^0.04
WN1	59.57 ± 1.11	86.27	1.36 ± 0.46	1.97	0.60 ± 0.01	0.87	7.04 ± 0.21	10.20	^1^0.06 ± 0.01, ^3^0.42 ± 0.02	^1^0.09, ^3^0.61
XC1	58.09 ± 9.52	91.42	3.51 ± 0.39	5.52	1.90 ± 0.13	2.99	ND		^5^0.04 ± 0.00	^5^0.06
XC2	46.99 ± 5.54	60.63	19.93 ± 2.59	25.72	5.04 ± 0.73	6.50	5.46 ± 1.56	7.05	^3^0.08 ± 0.04	^3^0.10
YJ	45.85 ± 6.21	70.72	13.31 ± 1.83	20.53	3.92 ± 0.91	6.05	ND	–	^2^1.20 ± 0.13, ^3^0.45 ± 0.06, ^5^0.10 ± 0.01	^2^1.85, ^3^0.69, ^5^0.15
YL2	68.63 ± 4.28	78.41	16.28 ± 1.35	18.60	2.17 ± 0.05	2.48	ND	–	^3^0.45 ± 0.02	^3^0.51
YX6	58.76 ± 11.59	73.02	15.14 ± 1.99	18.81	4.01 ± 0.01	4.98	ND	–	^2^2.31 ± 0.35, ^3^0.24 ± 0.02, ^4^0.01 ± 0.00	^2^2.87, ^3^0.30, ^4^0.01
ZT1	42.76 ± 2.51	67.04	9.18 ± 0.54	14.39	7.84 ± 0.28	12.29	ND	–	^1^2.88 ± 0.11, ^2^0.88 ± 0.09, ^3^0.16 ± 0.01, ^7^0.08 ± 0.01	^1^4.52, ^2^1.38, ^3^0.25, ^7^0.13
ZY3	49.04 ± 8.62	72.95	6.67 ± 0.54	9.92	4.31 ± 0.32	6.41	4.85 ± 0.09	7.22	^1^1.05 ± 0.24, ^3^0.58 ± 0.01, ^4^0.44 ± 0.02, ^5^0.18 ± 0.01, ^6^0.10 ± 0.01	^1^1.56, ^3^0.86, ^4^0.65, ^5^0.27, ^6^0.15
ZaZ4	40.01 ± 0.78	63.92	15.17 ± 0.64	24.24	7.21 ± 0.12	11.52	ND	–	^3^0.17 ± 0.01, ^7^0.03 ± 0.00	^3^0.27, ^7^0.05
ZeZ6	34.63 ± 0.11	70.75	5.32 ± 0.09	10.87	5.16 ± 0.40	10.54	2.24 ± 0.05	4.58	^1^0.48 ± 0.01, ^2^0.45 ± 0.02, ^3^0.36 ± 0.01, ^4^0.31 ± 0.03	^1^0.98, ^2^0.92, ^3^0.74, ^4^0.63
ZaZ6	50.94 ± 4.35	79.15	6.13 ± 0.28	9.52	4.34 ± 0.27	6.74	1.72 ± 0.01	2.67	^1^0.88 ± 0.13, ^3^0.24 ± 0.02, ^6^0.11 ± 0.01	^1^1.37, ^3^0.37, ^6^0.17
ZeZ9	32.80 ± 1.60	52.10	26.71 ± 0.18	42.42	2.66 ± 0.54	4.22	ND	–	^3^0.37 ± 0.02, ^5^0.32 ± 0.09, ^6^0.10 ± 0.01	^3^0.59, ^5^0.51, ^6^0.16
BJ7-2	76.20 ± 5.44	60.82	23.40 ± 1.43	18.68	6.90 ± 1.25	5.51	4.64 ± 0.42	3.70	^1^6.49 ± 0.84, ^2^6.96 ± 0.33, ^3^0.35 ± 0.05, ^5^0.18 ± 0.02, ^6^0.17 ± 0.02	^1^5.18, ^2^5.56, ^3^0.28, ^5^0.14, ^6^0.14
HF	35.46 ± 2.16	66.44	12.49 ± 1.20	23.40	3.44 ± 0.29	6.45	1.20 ± 0.22	2.25	^2^0.51 ± 0.08, ^3^0.23 ± 0.01, ^7^0.04 ± 0.01	^2^0.96, ^3^0.43, ^7^0.07
Mean	46.92	65.67	12.33	16.25	5.15	8.05	3.00	4.90	^1^1.67, ^2^1.53, ^3^0.30, ^4^0.21, ^5^0.15, ^6^0.11, ^7^0.04	^1^1.94, ^2^2.29, ^3^0.42, ^4^0.37, ^5^0.22, ^6^0.14, ^7^0.07
SD	16.87	0.10	8.06	0.09	2.67	0.05	1.78	0.03	^1^2.05, ^2^1.51, ^3^0.20, ^4^0.18, ^5^0.09, ^6^0.03, ^7^0.02	^1^0.02, ^2^0.02, ^3^0.002, ^4^0.004, ^5^0.003, ^6^0.001, ^7^0.001
CV (%)	35.96	14.13	65.40	54.45	51.81	55.35	59.28	65.95	^1^122.95, ^2^98.61, ^3^67.53, ^4^84.25, ^5^56.86, ^6^28.15, ^7^52.44	^1^92.53, ^2^76.03, ^3^54.25, ^4^108.11, ^5^90.91, ^6^71.43, ^7^142.86

Note: ND, not detected. Content: g·kg^−1^ DW, Pro, Proportion (%).

In addition to malic acid, three main organic acid components were also detected in Chinese cherry fruits. These three acids mainly included quinic acid, citric acid and succinic acid, with an average content of 12.33 g·kg^−1^ DW, 5.15 g·kg^−1^ DW, and 3.00 g·kg^−1^ DW, respectively (Table 2, Fig. 1C, D). Moreover, small amounts of tartaric acid (0.03 ∼ 6.49 g·kg^−1^ DW) and lactic acid (0.30 ∼ 6.96 g·kg^−1^ DW) were identified in Chinese cherries, along with trace amounts (0.01 ∼ 0.58 g·kg^−1^ DW) of oxalic, ascorbic, shikimic, maleic, and fumaric acid (Table 2). These components exhibited significant differences among experimental accessions,with the CV values ranging from 28.15 % (maleic acid) to 122.95 % (tartaric acid) (Fig. 1D). Furthermore, 10.02 % trace organic acids components were specifically determined in partial accessions (Fig. 1C). Small or trace amounts of organic acids not only are applied as important added ingredients in food flavor improvement, but also fulfill other flavor chemicals synthesis as potential procursors, showing their indispensable roles in the formation of food flavor (Li, Xie, Sun, Zhang, Liu & Liu, 2022). In addition, the CV of malic acid, with the highest proportion of organic acids in Chinese cherry fruits, was 35.96 % (Table 2, Fig. 1C, D). Our data suggest that organic acids possibly contribute more to flavor quality than soluble sugars. Consequently, identifying these organic acid compounds contributes greatly to a better understanding of the formation of organic acid in Chinese cherry fruits, and it also provides a theoretical reference for further improving food flavor quality dominanted by sugar and organic acid components.

### 3.3 Flavour evaluation of cultivated Chinese cherry fruits

Indicators such as SSC, TA, and the ratio of SSC to TA (SSC/TA) are commonly used to evaluate the sugar and acid flavor of fruit. The SSC/TA ratio, in particularly, can directly feflect the flavor of fruits (Wang et al., 2022). However, these indicators do not consider the specific contributions of individual sugar and acid components, which fails to provide a comprehensive understanding of the true sweet and acid flavor of the fruit. In this study, we conbined various soluble sugar and organic acid components to comprehensively evaluate the flavor of Chinese cherry fruits.

**Fig. 2:**
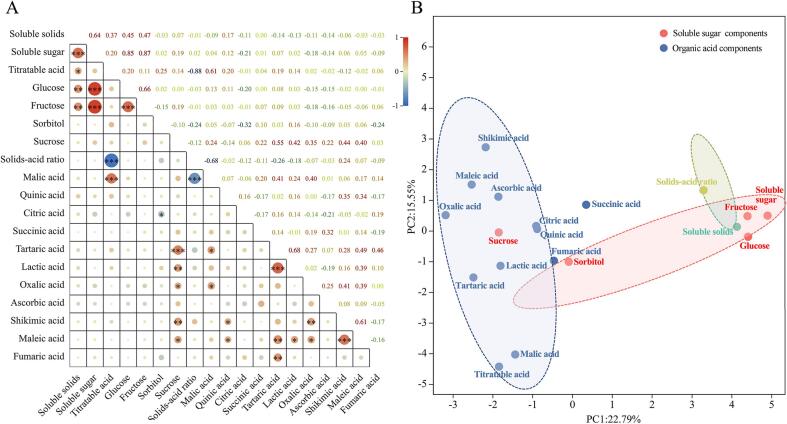
Correlation (A) and PCA (B) analysis of all evaluated variables for all sugar and organic acid-related traits in 38 cultivated Chinese cherry accessions. *, **, and *** indicate significance at 0.05, 0.01, and 0.001 level, respectively.

To better understand the relationship between fruit flavor variables of Chinese cherries, a correlation analysis was conducted (Fig. 2A). Soluble solid contents showed significantly positive correlations with soluble sugars (0.64^***^), glucose (0.45^**^), and fructose (0.47^**^), which is consistent with previous results in Chinese cherry (Wang et al., 2022). Highly significant positive correlations were also observed among soluble sugar, glucose, and fructose (*r* = 0.66^***^ ∼ 0.87^***^), similar to reports in blueberry (Zhang et al., 2020) and apple (Li et al., 2021). The glucose content exihibited relatively high correlation with the soluble sugar contents between the two prodominant soluble sugars. Among all the identified organic acids, malic acid exihited the highest correlation (0.61^***^) with titratable acid content. Meanwhile, a highly significant negative correlation was observed between SSC/TA and malic acid (−0.68^***^). This further suggests that malic acid has a greater effect on the falvor of Chinese cherry fruits than other organic acids. The content of malic acid largely contributes to acid formation, as observed in apricot, where the perception of acidity is most strongly correlated with malate (Baccichet et al., 2022).

Principal component analysis was also performed to evaluate change in flavor indicators in Chinese cherry fruits (Fig. 2B). The results showed that all variables could be distinguished by two principal components, PC1 and PC2, which explained 22.79 % and 15.55 % of the total discrepancy, respectively. PC1 mainly encompassed soluble sugars, soluble solids, SSC/TA, fructose, and glucose. Representative variables for PC2 included titratable acid, malic acid, and other minor acids. Based on the aforementioned results, SSC/TA, titratable acid, malic acid, soluble solids, soluble sugars, fructose, and glucose content were considered key as indicators for flavor evaluation in Chinese cherry.

### 3.4 Comparison of sugar and organic acid between cultivated and wild Chinese cherry

**Fig. 3:**
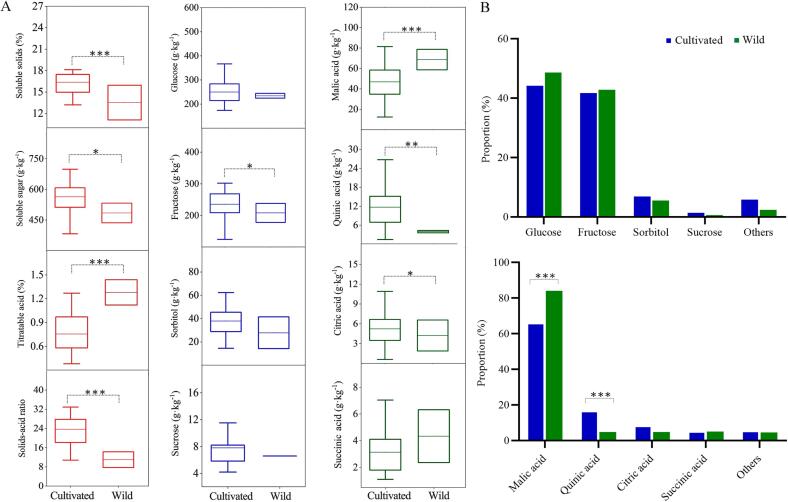
Comparison of distribution (A) and proportion (B) of sugar and organic acid contents between cultivated and wild Chinese cherry fruits. The horizontal lines in the interior of the box are the median values. The box indicates the distribution for 50% of the data. Approximately 99% of the data falls inside the whiskers. *, **, and *** indicate significance at 0.05, 0.01, and 0.001 level, respectively.

Wild Chinese cherry has the ability to adapt to harsh natural conditions and serve as a crucial genetic source for improving and innovating cherry germplasm (Chen et al., 2020). To explore the changes in soluble sugar and acid during the domestication process, we analyzed sugar and acid components and contents of two representative wild Chinese cherry accessions (Fig. 3A). Overall, cultivated cherries exhibited significangly higher sugar content (563.52 g·kg^−1^ DW for soluble sugar, 16.37 % for SSC) compared with wild cherries (484.34 g·kg^−1^ DW for soluble sugar, 13.53 % for SSC). The titratable acid contents in wild cherries were approximately twice as much (1.28 %) as those in cultivated cherries (0.76 %) (*p* < 0.001). The SSC/TA ratio ranged from 10.77 to 42.32 in cultivated cherries and from 7.71 to 14.24 in wild cherries, with a mean value of 23.62 in cultivated cherries, which was over 2.2-fold higher than the mean value of 10.98 in wild cherries. These results indicate that the fruit flavor of cultivated Chinese cherry has undergone domestication, leading to increased sugar content and decreased acidity.

Specifically, the mean fructose content in cultivated cherry was 235.88 g/kg DW, significantly higher than that in wild cherry (208.43 g·kg^−1^ DW) (*p* < 0.05) (Fig. 3A). The average contents of glucose, sucrose, and sorbitol were slightly higher in cultivated cherry compared with wild cherry, but these differences were not statistically significant (*p* > 0.05). Therefore, an increasing trend in fructose content was observed during the domestication process of Chinese cherry. The mean malic acid content in wild cherry was 73.63 g·kg^−1^ DW (range: 58.56 ∼ 88.69 g·kg^−1^ DW), significantly higher than that in cultivated cherry (mean: 46.92 g·kg^−1^ DW, range: 12.39 ∼ 81.39 g·kg^−1^ DW). Similar differences in acidity between cultivated and wild cherry were also observed for other major acids such as citric acid and quinic acid (*p* < 0.01). Furthermore, the range of variation in most sugar and organic acid components was much wider in cultivated cherry compared with wild cherry.

Traditionally, the domestication of fruit trees has been associated with an increase in fruit brix and a decrease in acidity, a phenomenon known as the “Hitchhiking effect” (Wang et al., 2018). It has been reported that cultivated apples exhibit domestication syndrome, characterized by larger fruit and higher soluble sugar content compared to their wild relatives (Parker, López, Petersen, Anaya, Cubilla-Rios & Potter, 2010). Our previous study indicated that cultivated Chinese cherry originates from wild cherry in the Longmenshan Fault zone (Zhang et al., 2021). In this study, we observed significant increases in SSC and fructose contents in cultivated cherry, along with substantial decreases in total acid and major components compared with wild cherry. Additionally, there were no significant changes in the proportions of different sugar components among the total sugars (Fig. 3B). In contrast, the proportion of malic acid decreased significantly from wild to cultivated cherry, while the proportions of citric acid and other acids increased significantly. These results suggest that the most interesting aspect of the domestication history of Chinese cherry fruits is the significant reduction in acid, particularly malic acid. This finding is in accordance with previous results in citrus (Wang et al., 2018) and contributes to improved taste.

### 3.5 Rating of fruit flavor of Chinese cherry

The SSC/TA ratio is a valuable indictor of fruit ripeness and plays a crucial in determining fruit flavor (Nowicka et al., 2019; Wang, Zhang, Zan, Gao, Tian & Meng, 2021). Based on the range of SSC/TA values, we classified fruit flavor into four categories: sour, sour–sweet, sweet–sour, and sweet (Table S2). The overall range of SSC/TA values ranged from 7.71 to 42.32 (Table S1), similar to sweet cherry (Ballistreri et al., 2013, Gonçalves et al., 2021), but much higher than that of sour cherry (Sokół-Łętowska, Kucharska, Hodun & Gołba, 2020). We further compared this classification with the taste ratings. The results of the taste evaluation showed that all the examined materials contained varying levels of sourness rather than pure sweetness. Likewise, there were significant differences between taste evaluation and grading evaluation, as confirmed by the cardinality test (*p* ＜ 0.001^**^) (Table S3).

Recongnizing the disparities between taste evaluation and grading evaluation, we conducted a digital quantification of fruit flavor-related traits. Through correlation and PCA analysis, we identified seven key indicators as flavor indicators of Chinese cherry: solids-acid ratio, total soluble solid, soluble sugar, glucose, fructose, titratable acid, and malic acid. All these indicators followed a normal distribution (Fig. S2). Each indicator was then categorized into five ratings based on the 10th, 30th, 70th, and 90th percentiles of the normal distribution curve (Table 3). The SSC/TA ratio primarily reflected the overall fruit taste, with higher values and grades indicating more intense fruit flavors. Previous studies have proposed grading criteria for major fruit quality traits, such as TSS, SS, and TA content of Chinese cherry (Wang et al., 2022). In this study, we screened key evaluation indicators and developed specific grading criteria for Chinese cherry fruit flavor quality, providing a theoretical basis for defining breeding objectives for Chinese cherry fruit.

**Table 3:** Detailed rating in fruit flavor of Chinese cherry.

Indexs	Rating (Solids-acid ratio)				
	1 (＜15.0)	2 (15.0–20.0)	3 (20.0–25.0)	4 (25.0–30.0)	5 (≥30.0)
Total soluble solid / %	＜12.0	12.0–15.0	15.0–18.0	18.0–21.0	≥21.0
Titratable acid / %	＜0.3	0.3–0.6	0.6–0.9	0.9–1.2	≥1.2
Soluble sugar / g·kg^−1^ DW	＜400.0	400.0–500.0	500.0–600.0	600.0–700.0	≥700.0
Glucose / g·kg^−1^ DW	＜150.0	150.0–210.0	210.0–270.0	270.0–330.0	≥330.0
Frutcose / g·kg^−1^ DW	＜160.0	160.0–210.0	210.0–260.0	260.0–310.0	≥310.0
Malic acid / g·kg^−1^ DW	＜25.0	25.0–40.0	40.0–55.0	55.0–60.0	≥60.0

Sugars and organic acids composition is one of the key factors that determine the quality of fruit flavor (Blando & Oomah, 2019). However, during the fruit development, the metabolism of soluble sugars and organic acids is a very complex physiological process, which is significantly affected by genotypes, tree age and environment, and in their metabolism process, soluble sugars and organic acids are often converted to each other (Colantonio et al., 2020). All of these facts demonstrate the difficulty in conducting the flavor evaluation based on the sugar and organic acids composition. In this study, all examined accessions were planted in the same orchad, with the same tree age, thereby our results partially reflect the effects of genotypes on the sugars and organic acids metabolism. The flavor indicators involving the sugars and organic acids can provide valuable reference for the fruit flavor improvement (Lin et al., 2023). Meantime, the contents and proportions of sugars and organic acids also offer the guidance for the flavor qualities evulation of other foods as well as the food flavor improvement in food industry.

Additionally, trace sugars and organic acids components also contribute to the flavor (Lin et al., 2023). Except for sugars and organic acids, many other second metabolites, such as amino acids, flavonoids and alkaloids, are also crucial contributors to flavor (Cao et al., 2022). Since the flavor is collectively determined by sugars, acids and other metabolites and is subject to the changes and alteration within and among these chemicals (Shu et al., 2022), it is necessary to ulitize omics-based methods, such as metabolomics, to carry out more refine identification of the flavor-related chemical components.

## 4 Conclusion

This study presents the composition of sugar and organic acid in Chinese cherry fruits for the first time. The main soluble sugar in Chinese cherry fruit are glucose and fructose, with the proportions varying depending on the variety. Among the various organic acids, malic acid plays a predominant role. Correlation and principal component analyses revealed that the key indicators for evaluating the flavor quality of Chinese cherry include solids-acid ratio, soluble solids, soluble sugars, glucose, fructose, titratable acid, and malic acid. The total acid and acid composition in cultivated Chinese cherry were significantly lower than those in wild cherry, whereas the fructose and total soluble sugar content in cultivated cherries were significantly higher than those in wild cherry. The domestication process of sugars and acids, particularly the significant reduction in acids, especially malic acid, stands out as the most distinctive feature of Chinese cherry fruit. Grading criteria were established for these seven evaluation indicators, providing a means to assess the sweet and sour flavour of Chinese cherry fruit. The results of this study will serve as a foundation for fine mapping of genes associated with superior quality-related traits in Chinese cherry germplasm resources and contribute to further genetic improvement of Chinese cherry flavor quality.

**Funding**

This work was financially supported by Cherry Resources Sharing and Service Platform of Sichuan Province, Natural Science Foundation of Sichuan Province (2023NSFSC0158), Chengdu Technological Innovation Research and Development Project (2022YF05-01017-SN), Tianfu Talent Project of Chengdu City (2021-CF02-0162396-RC-4096), and The Project of Rural Revitalization Research Institute in Tianfu New Area of Sichuan Province (XZY1-04).

## CRediT authorship contribution statement

**Jingting Zhou:** Methodology, Investigation, Formal analysis, Data curation, Writing – original draft. **Shuaiwei Yang:** Methodology, Investigation, Formal analysis. **Yan Ma:** Investigation, Formal analysis. **Zhenshan Liu:** Investigation. **Hongxia Tu:** Investigation. **Hao Wang:** Data curation. **Jing Zhang:** Data curation. **Qing Chen:** Methodology, Software. **Wen He:** Software. **Mengyao Li:** Software. **Yuanxiu Lin:** Resources. **Yunting Zhang:** Resources. **Zhiwei Wu:** Project administration. **Yong Zhang:** Validation. **Ya Luo:** Visualization. **Haoru Tang:** Supervision. **Yan Wang:** Supervision, Funding acquisition, Writing – review & editing. **Xiaorong Wang:** Supervision, Conceptualization, Funding acquisition, Writing – review & editing.

## Declaration of Competing Interest

The authors declare that they have no known competing financial interests or personal relationships that could have appeared to influence the work reported in this paper.

## Data Availability

Data will be made available on request.
