# Supplementary material for: Soluble sugar and organic acid composition and flavor evaluation of Chinese cherry fruits
Source: Food Chem X. 2023 Oct 21;20:100953. doi: 10.1016/j.fochx.2023.100953 (PMC10622630; doi:10.1016/j.fochx.2023.100953)
Supplement: Supplementary data 1 [file mmc1.docx]

**Table S1**

Description of characteristics of Chinese cherry fruits in this study.

| Code | Accession | Type | Locality  (City, Province) | Maturity  date^1^ | Fruit  size (g) ^2^ | Peel color^3^ | Fruit shape^4^ | SSC (%)^5^ | TA (%)^6^ | SSC/TA^7^ | Sensory flavor | |
| --- | --- | --- | --- | --- | --- | --- | --- | --- | --- | --- | --- | --- |
|  |  |  |  |  |  |  |  |  |  |  | Rating | Score |
| 1 | ZaZ8 | Landrace | Zaozhuang, Shandong | 21 to 25 Apr. | 2.23-2.62 | Red | Near round | 15.37±0.30 | 0.68±0.01 | 25.54 | Sour-sweet | 3.28 |
| 2 | BJ2 | Landrace | Bijie, Guizhou | 18 to 25 Apr. | 2.91-3.91 | Red | Near round | 14.87±0.83 | 0.61±0.05 | 24.38 | Sweet-sour | 6.12 |
| 3 | BJ4 | Landrace | Bijie, Guizhou | 7 to 23 Apr. | 4.30-5.16 | Red | Near round | 16.40±0.41 | 0.59±0.01 | 27.80 | Sweet-sour | 6.85 |
| 4 | BJ6 | Landrace | Bijie, Guizhou | 18 to 25 Apr. | 2.64-4.09 | Red | Near round | 17.80±0.21 | 0.76±0.06 | 23.42 | Sweet-sour | 6.67 |
| 5 | BJ7 | Landrace | Bijie, Guizhou | 12 to 23 Apr. | 2.84-4.07 | Red | Near round | 17.23±0.31 | 0.58±0.04 | 29.71 | Sweet-sour | 7.01 |
| 6 | FM2 | Landrace | Kunming, Yunnan | 18 to 25 Apr. | 2.90-3.86 | Red | Near round | 17.83±0.37 | 0.60±0.04 | 29.72 | Sweet-sour | 7.12 |
| 7 | GY1 | Landrace | Guiyang, Guizhou | 21 to 25 Apr. | 3.31-4.55 | Orange red | Near round | 17.20±1.23 | 0.92±0.04 | 18.70 | Sour-sweet | 2.95 |
| 8 | GY2 | Landrace | Guiyang, Guizhou | 18 to 25 Apr. | 2.48-2.68 | Red | Near round | 18.12±0.99 | 0.63±0.03 | 28.76 | Sweet-sour | 6.89 |
| 9 | HC | Landrace | Panzhihua, Sichuan | 12 to 25 Apr. | 2.25-2.63 | Orange red | Oblate | 13.68±1.06 | 1.27±0.08 | 10.77 | Sour-sweet | 2.61 |
| 10 | HeF | Cultivar | Ya'an, Sichuan | 22 to 25 Apr. | 3.36-3.37 | Red | Oblate | 18.10±1.33 | 0.79±0.11 | 22.91 | Sour-sweet | 2.82 |
| 11 | HZ1 | Landrace | Bijie, Guizhou | 12 to 25 Apr. | 2.40-3.16 | Red | Near round | 15.05±0.23 | 0.56±0.03 | 26.88 | Sweet-sour | 5.78 |
| 12 | HZZ | Cultivar | Chongqing | 12 to 25 Apr. | 2.94-4.02 | Red | Near round | 16.70±0.32 | 0.66±0.11 | 28.33 | Sweet-sour | 6.11 |
| 13 | JY4 | Landrace | Chengdu, Sichuan | 18 to 25 Apr. | 4.24-5.26 | Vermilion on yellow ground | Reniform | 16.75±0.36 | 0.66±0.12 | 25.38 | Sour-sweet | 4.62 |
| 14 | LQ | Landrace | Chengdu, Sichuan | 18 to 25 Apr. | 5.09-7.67 | Vermilion on yellow ground | Ellipse | 14.80±0.74 | 0.58±0.06 | 25.52 | Sour-sweet | 4.77 |
| 15 | LYg | Landrace | Luoyang, Henan | 14 to 20 Apr. | 2.27-2.69 | Orange red | Heart | 13.95±0.68 | 0.62±0.02 | 22.50 | Sour-sweet | 3.65 |
| 16 | LYi5 | Landrace | Linyi, Shandong | 18 to 25 Apr. | 1.60-2.59 | Orange red | Near round | 14.40±0.22 | 0.65±0.02 | 22.15 | Sour-sweet | 4.07 |
| 17 | MY3 | Landrace | Panzhihua, Sichuan | 18 to 25 Apr. | 2.23-2.97 | Orange red | Reniform | 17.60±0.35 | 0.97±0.16 | 18.14 | Sour-sweet | 3.26 |
| 18 | MY5 | Landrace | Panzhihua, Sichuan | 18 to 25 Apr. | 2.36-2.83 | Orange red | Reniform | 17.47±0.27 | 1.06±0.05 | 16.48 | Sour-sweet | 3.31 |
| 19 | MZ3 | Landrace | Honghe, Yunnan | 18 to 25 Apr. | 2.75-3.74 | Orange red | Oblate | 14.97±0.38 | 1.23±0.09 | 12.17 | Sour | 1.56 |
| 20 | NZH | Landrace | Chongqing | 1 to 14 Apr. | 2.27-3.88 | Orange red | Reniform | 15.30±0.43 | 0.74±0.04 | 20.68 | Sour-sweet | 3.64 |
| 21 | PD3 | Landrace | Anshun, Guizhou | 21 to 25 Apr. | 2.81-4.09 | Orange red | Near round | 14.85±0.74 | 0.92±0.22 | 16.14 | Sour | 1.88 |
| 22 | PJHH | Landrace | Chengdu, Sichuan | 12 to 23 Apr. | 2.29-2.20 | Red | Near round | 16.78±0.40 | 0.51±0.06 | 32.90 | Sweet-sour | 7.17 |
| 23 | SP4 | Landrace | Honghe, Yunnan | 12 to 23 Apr. | 3.20-3.33 | Orange red | Oblate | 16.98±0.30 | 1.05±0.05 | 14.27 | Sour-sweet | 2.96 |
| 24 | TH2 | Landrace | Fuyang, Anhui | 12 to 25 Apr. | 2.22-2.89 | Red | Near round | 15.30±0.88 | 0.47±0.02 | 32.55 | Sweet-sour | 7.26 |
| 25 | WN1 | Landrace | Bijie, Guizhou | 12 to 24 Apr. | 2.77-4.07 | Red | Near round | 16.00±0.75 | 0.62±0.09 | 25.81 | Sour-sweet | 4.56 |
| 26 | XC1 | Landrace | Xichang, Sichuan | 17 to 25 Apr. | 2.83-3.40 | Vermilion on yellow ground | Oblate | 17.72±0.90 | 1.16±0.11 | 15.28 | Sour-sweet | 3.02 |
| 27 | XC2 | Landrace | Xichang, Sichuan | 18 to 21 Apr. | 1.45-2.73 | Yellow | Oblate | 18.02±0.28 | 1.06±0.03 | 17.00 | Sour-sweet | 3.26 |
| 28 | YJ | Landrace | Ya'an, Sichuan | 7 to 23 Apr. | 2.90-3.26 | Red | Oblate | 13.20±0.54 | 0.52±0.01 | 25.38 | Sour-sweet | 4.78 |
| 29 | YL2 | Landrace | Kunming, Yunnan | 18 to 25 Apr. | 2.22-3.15 | Orange red | Oblate | 16.43±0.19 | 1.09±0.06 | 15.07 | Sour | 2.11 |
| 30 | YX6 | Landrace | Yuxi, Yunnan | 18 to 23 Apr. | 3.16-4.03 | Orange red | Near round | 17.65±0.20 | 0.72±0.08 | 24.51 | Sour-sweet | 4.22 |
| 31 | ZT1 | Landrace | Zaotong, Yunnan | 18 to 23 Apr. | 3.43-4.79 | Red | Near round | 15.17±0.76 | 0.61±0.01 | 24.87 | Sweet-sour | 5.68 |
| 32 | ZY3 | Landrace | Ziyang, Sichuan | 18 to 23 Apr. | 3.11-5.08 | Red | Near round | 16.20±0.34 | 0.59±0.04 | 27.46 | Sweet-sour | 6.08 |
| 33 | ZaZ4 | Landrace | Zhengzhou, Henan | 12 to 21 Apr. | 1.33-1.54 | Black purple | Heart | 27.53±1.49 | 1.17±0.05 | 23.53 | Sweet-sour | 5.44 |
| 34 | ZeZ6 | Landrace | Zhengzhou, Henan | 16 to 21 Apr. | 2.61-2.70 | Red | Near round | 13.36±0.82 | 0.73±0.05 | 31.07 | Sweet-sour | 6.51 |
| 35 | ZaZ6 | Landrace | Zaozhuang, Shandong | 20 to 21 Apr. | 2.41-2.63 | Vermilion on yellow ground | Heart | 13.60±0.24 | 0.78±0.03 | 23.45 | Sweet-sour | 5.12 |
| 36 | ZeZ9 | Landrace | Zhengzhou, Henan | 12 to 21 Apr. | 1.80-2.18 | Red | Near round | 16.08±0.19 | 0.38±0.04 | 42.32 | Sweet-sour | 7.38 |
| 37 | BJ7-2 | Landrace | Bijie, Guizhou | 21 to 23 Apr. | 2.15-3.13 | Vermilion on yellow ground | Oblate | 16.92±0.40 | 1.12±0.03 | 15.11 | Sour | 1.79 |
| 38 | HF | Cultivar | Chongqing | 7 to 22 Apr. | 4.66-5.57 | Red | Heart | 16.77±0.75 | 0.54±0.01 | 31.06 | Sweet-sour | 5.82 |
| 39 | Wild1 | Wild | Bazhong, Sichuan | 23 to 25 Apr. | 0.97-1.31 | Vermilion on yellow ground | Near round | 11.10±0.23 | 1.44±0.09 | 7.71 | Sour | 1.23 |
| 40 | Wild2 | Wild | Bijie, Guizhou | 24 to 25 Apr. | 0.59-0.91 | Orange red | Near round | 15.95±0.34 | 1.12±0.06 | 14.24 | Sour | 1.44 |

Note: Fruit-related traits were measured based on the phenotyping protocol of Chinese cherry (Wang et al., 2022). 1, 2, 3 and 4 were evaluated during three years in the plant materials. 5, 6, 7 and 8 represent the results for year 2022. SSC, soluble solid content. TA, titratable acid. SSC/TA, the ratio of SSC to TA.

**Table S2**

Cardinality test for sensory evaluation and grade rating.

|  |  | Sensory evaluation | | |  | Total | χ^2^ | *p* |
| --- | --- | --- | --- | --- | --- | --- | --- | --- |
|  |  | Sweet-sour | Sour | Sour-sweet |  |  |  |  |
| Grade rating | Sweet | 100.00% | 0.00% | 0.00% |  | 3 | 23.042 | 0.001** |
|  | Sweet-sour | 66.67% | 0.00% | 33.33% |  | 18 |  |  |
|  | Sour | 0.00% | 60.00% | 40.00% |  | 5 |  |  |
|  | Sour-sweet | 14.29% | 21.43% | 64.29% |  | 14 |  |  |
| Total | | 42.50% | 15.00% | 42.50% |  | 40 |  |  |

Note: **p* < 0.05, ***p* < 0.01.

**Table S3**

General type in fruit flavor of Chinese cherry.

| Indexs | General type (Solids-acid ratio) | | | |
| --- | --- | --- | --- | --- |
|  | Sour (＜15.0) | Sour-sweet (15.0-23.5) | Sweet-sour (23.5-32.0) | Sweet (≥32.5) |
| Total soluble solid / % | 11.1-17.0 | 13.6-18.1 | 13.2-27.6 | 15.3-16.8 |
| Titratable acid / % | 1.1-1.44 | 0.6-1.2 | 0.4-1.2 | 0.4-0.5 |
| Soluble sugar / g·kg^-1^ DW | 381.3-580.6 | 473.6-673.0 | 428.4-697.6 | 589.1-698.6 |
| Glucose / g·kg^-1^ DW | 187.8-262.3 | 204.5-366.5 | 173.5-344.8 | 261.9-343.7 |
| Fructose / g·kg^-1^ DW | 124.1-258.2 | 189.6-291.2 | 143.0-294.8 | 212.5-286.4 |
| Malic acid / g·kg^-1^ DW | 55.6-88.7 | 25.8-81.4 | 13.0-59.6 | 24.7-38.5 |
| Representative landrace | HC | HeF | HF | PJHH |


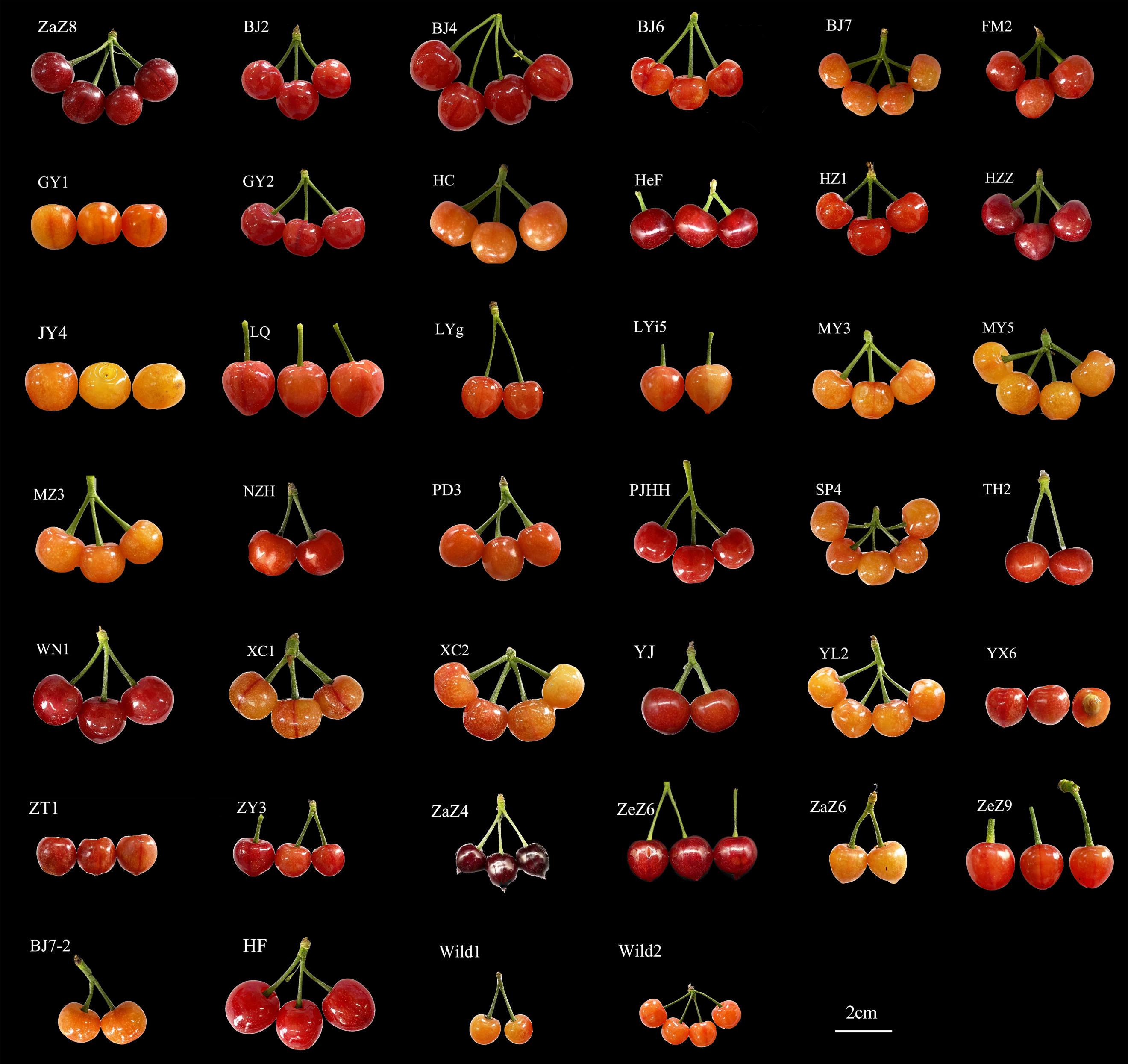


**Fig. S1.** Fruit phenotypes of forty Chinese cherry accessions in this study.


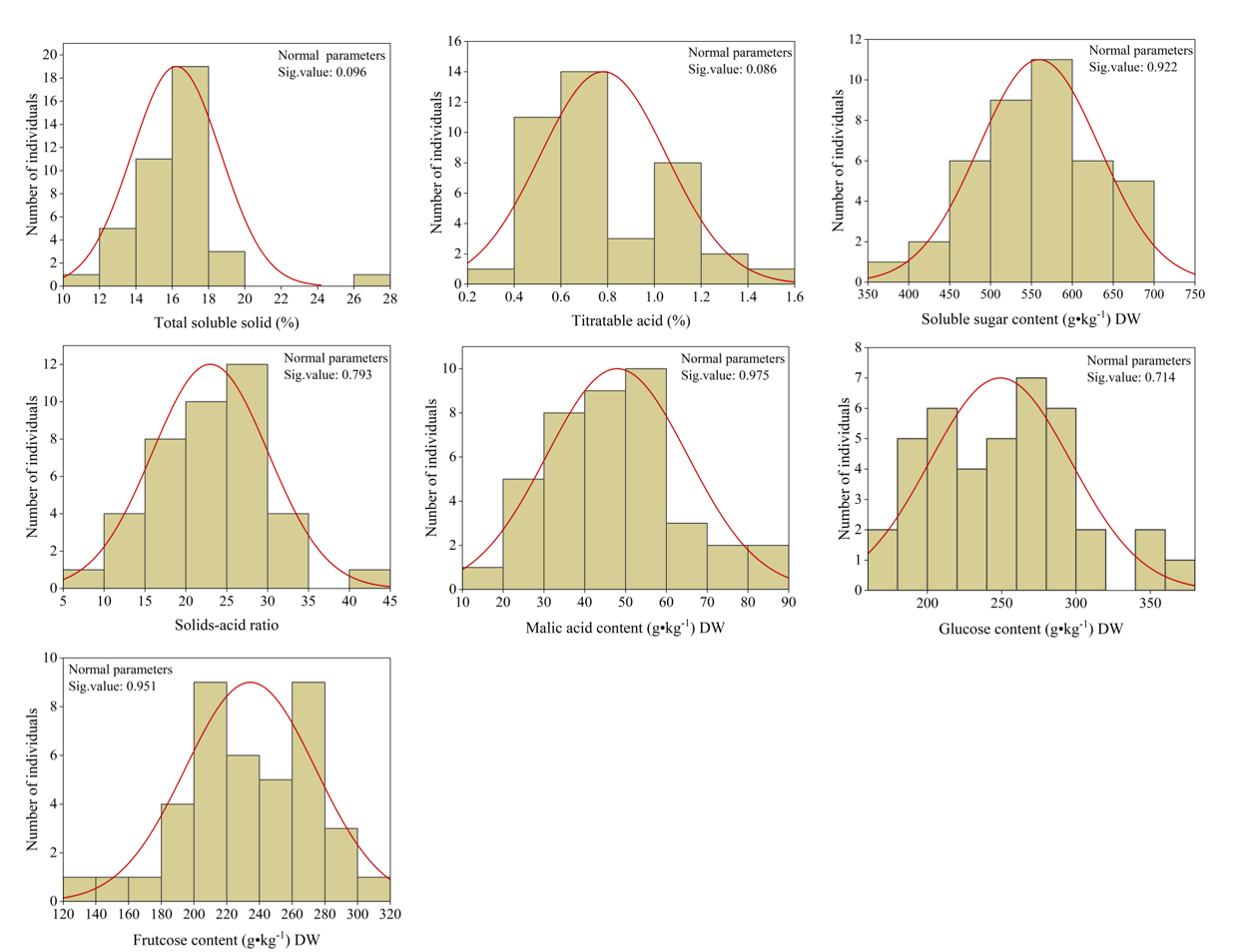


**Fig. S2.** Normal distribution of seven fruit sugar and organic acid indices in Chinese cherry fruits.

Note: Sig. value, significance. Sig. value ≥ 0.05 indicates a normal distribution.

**References:**

Wang, Y., Hu, G., Liu, Z., Zhang, J., Ma, L., Tian, T., Wang, H., Chen, T., Chen, Q., He, W., Yang, S., Lin, Y., Zhang, Y., Li, M., Zhang, Y., Luo, Y., Tang, H., & Wang, X. (2022). Phenotyping in flower and main fruit traits of Chinese cherry [Cerasus pseudocerasus (Lindl.) G.Don]. *Scientia Horticulturae*, *296*, 110920.https://doi.org/10.1016/j.scienta.2022.110920
